# Supplementary material for: Aggregation-Induced Ignition of Near-Infrared Phosphorescence of Non-Symmetric [Pt(C^N*N’^C’)] Complex in Poly(caprolactone)-based Block Copolymer Micelles: Evaluating the Alternative Design of Near-Infrared Oxygen Biosensors
Source: Biosensors (Basel). 2022 Aug 28;12(9):695. doi: 10.3390/bios12090695 (PMC9496585; doi:10.3390/bios12090695)
Supplement: Supplementary file 1 [file biosensors-12-00695-s001.zip › biosensors-1852722-supplementary materials.pdf]

biosensors

MDPI

Supplementary Material for the Article

Aggregation-Induced Ignition of Near-Infrared Phosphorescence of Non-Symmetric [Pt(C^N*N′^C′)] Complex in Poly(caprolactone)-based Block Copolymer Micelles: Evaluating the Alternative Design of Near-Infrared Oxygen Biosensors

Nina A. Zharskaia [1], Anastasia I. Solomatina [1], Yu-Chan Liao [2], Ekaterina E. Galenko [1], Alexander F. Khlebnikov [1], Pi-Tai Chou [2,*], Pavel S. Chelushkin [1,*] and Sergey P. Tunik [1]

[1] Institute of Chemistry, St. Petersburg State University, Universitetskii av., 26, 198504 St. Petersburg, Russia
[2] Department of Chemistry, National Taiwan University, National Taiwan University, No. 1, Sec. 4, Roosevelt Rd., Taipei 10617, Taiwan
* Correspondence: chop@ntu.edu.tw (P.-T.C.); p.chelushkin@spbu.ru (P.S.C.)

Part 1. Materials and Methods

1. General comments

N,*N*-dimethylformamide (DMF; analytical grade; "Ekos-1", Russia) was used as received. Type 1 water was obtained using a "Simplicity" water purification system ("Merck Millipore", USA). Phosphate Buffered Saline (PBS) was prepared from tablets ("Amresco Inc.", USA). Isotonic PBS solutions were prepared by dissolving 1 tablet in 100 mL of type 1 water; 10-fold concentrated stock solutions by dissolving 1 tablet in 10 mL of type 1 water. Calf serum (CS) and fetal bovine serum (FBS) were purchased from "Biolot", Russia, and "Thermo Fisher Scientific", USA, respectively. Synthesis and characterization of platinum(II) [Pt(C^N*N′^C′)] complex **Pt1** (Scheme 1) was described in detail in our previous publication [S1].

The preparative centrifugations were performed in 1.5 mL polypropylene capped tubes at 15,000 rpm (ca. 20,000 g) using "Sigma 2-16P" preparative centrifuge equipped with a "12148-H" fixed-angle rotor ("Sigma", Germany). ^{1}H NMR spectra were recorded using a "Bruker Avance 400" spectrometer ("Bruker", Germany); all spectra were recorded in DMSO-d_6 at room temperature. Analytical gel permeation chromatography (GPC) was performed on "Prominence LC-20AD" ("Shimadzu", Japan) chromatograph equipped with refractomentric detector and "PLgel MIXED-C" column (300 × 7.5 mm, 5 μm particles, linear molecular weight range up to 2000 kg/mol based on polystyrene, "Agilent Technologies", Netherlands). Runs were performed in THF at 40°C and 1.0 mL/min flow rate, P = 4.2–4.3 MPa. Block copolymer solutions (3 mg/mL) were filtered through 0.22 μm PTFE filters. Mass-average molar masses (M_w) and dispersities ($Đ = M_w/M_n$) were calculated using the "Shimadzu LCsolution" software. Cubic calibration curve was built using a set of polystyrene standards (500–250,000 g/mol).

*2. Estimation of micelles loading by **Pt1** with UV/Vis absorption spectroscopy*

Weight fractions ('loadings') of **Pt1** in **Pt1@PCL-*b*-PEG** micelles (expressed as wt.% of **Pt1**) were calculated from UV/Vis absorption spectra recorded in DMF. Both components of the nanoparticles are independently soluble in this solvent, and thus the hydrophobic interactions responsible for micelle formation can be expected to be substantially eliminated and nanoparticles disintegrate. The resulting absorption spectra of the dissolved nanoparticles can be treated as a linear combination of the individual absorption spectra. Molar concentration of the complex **Pt1** was calculated from its absorbance at 381 nm using the Beer–Lambert–Bouguer law (A = εlc) and molar absorption coefficients (ε) of the starting complex were determined independently in DMF (the calibration curve of absorption of **Pt1** at 381 nm in the presence of 0.25 mg/mL **PCL-*b*-PEG** is presented in Figure S2).

3. Dynamic light scattering (DLS)

DLS experiments were performed on a "PhotoCor-Complex" instrument ("PhotoCor Instruments Inc.", Russia) equipped with a real-time correlator (288 channels, minimal τ = 20 ns). Laser (λ = 654 nm) was used as an excitation source; the experiments were carried out at scattering angles (ϑ) ranging from 30° to 140° at a temperature of 25 ± 0.1 °C. DynaLS software was used to fit the autocorrelation functions of scattered light intensity $G^{(2)}(t) = \langle I(t_0)I(t_0+t)\rangle/\langle I(t_0)\rangle^2$. Distributions $I(\tau)$ of scattered light intensities by relaxation times τ were calculated according to the relation: $G^{(1)}(t) = \int E(\tau)\, e^{-t/\tau} d\tau$, where $G^{(1)}(t)$ is related to $G^{(2)}(t)$ by Siegert relation $G^{(2)}(t) = B + \beta\left|G^{(1)}(t)\right|^2$, B is baseline, and β is coherence factor.

Translational diffusion coefficients D at fixed concentrations were calculated from the slope of this line according to the following relationship: $1/\tau = Dq^2$. The diffusion coefficients D_0 were determined by extrapolation of $D(c)$ dependence to infinite dilution according to equation: $D(c) = D_0(1 + c2A_2M)$. Hydrodynamic radii R_h were calculated using the Stokes – Einstein equation:

$$R_h = k_B T/(6\pi\eta_0 D_0), \quad \text{(S1)}$$

where k_B is the Boltzmann's constant; T is the absolute temperature, and η_0 is the solvent viscosity.

4. Photophysical experiments

All photophysical measurements were carried out in either DMF or aqueous dispersions based on type 1 water. UV/Vis spectra were recorded using a "Shimadzu UV-1800" spectrophotometer ("Shimadzu", Japan) in "QS" 10 mm high precision absorption cells ("Hellma Analytics", Germany). Luminescence emission and excitation spectra in aqueous dispersions of polymer micelles were recorded on a "FluoroMax-4" spectrofluorometer ("JY Horiba Inc.", Japan) in "QS" 10x10 mm high precision luminescence cells ("Hellma Analytics", Germany) at concentrations of 0.2 mg/mL.

Lifetimes in aqueous dispersions were determined by the TCSPC method using an home-build setup consisting of "DTL-375QT Basic" pulsed laser ("Laser-export Co. Ltd.", Russia; 355 nm), "Tektronix" oscilloscope (DPO2012B, band width 100 MHz), "Monoscan-2000" scanning monochromator ("Ocean Optics", USA; interval of wavelengths 1 nm), "FASTComTec" multiple-event time digitizer (model: MCS6A1T4), and "Hamamatsu" photon counting head (model: H10682-01). The nanosecond time-resolved measurements were performed by time-correlated single-photon counting technique (TCSPC, OB900L lifetime spectrometer, Edinburgh) including the EPL-375 laser as an excitation source. A "FireStingO2" oxygen meter ("PyroScience", Germany) equipped with an "OXROB10" oxygen probe and "TDIP15" temperature sensor) was used to determine the partial pressure of molecular oxygen in aqueous dispersions. The lifetimes raw data were fit using the "Origin 2015" software. Lifetime dependences on O_2 partial pressure ($p(O_2)$) were linearized using Stern-Volmer equation:

$$\frac{\tau_0}{\tau} = 1 + K_{SV}p(O_2) = 1 + \tau_0 K_Q p(O_2), \quad \text{(S2)}$$

where τ_0 and τ are lifetimes in the absence and presence of a quencher (O_2), respectively; K_{SV} – Stern-Volmer constant, and K_Q – quenching constant.

Quantum Yield (QY) measurements were performed by a comparative method using the sample **Pt1@PCL-*b*-PEG** based on non-purified sample #2 of **PCL-*b*-PEG** (instead, the resulting micelles were purified from the precipitate by preparative centrifugation). Light emission diode was used as an excitation source and air-saturated solutions of $[Ru(bpy)_3][PF_6]_2$ in water (QY = 0.040) was used as reference. In these experiments, an enhanced scattering was observed due to colloidal nature of the micellar dispersions. As a result, the measurement uncertainty appeared to be higher than that for conventional QY measurements in solutions; we estimate it from 20 to 30 %.

Measurements of all the photophysical data were repeated at least in triplicate. In all cases where it was possible, the measurements were performed from independent micellar preparations. In the case of single micelle preparation, we estimate the measurements error to be of 5 %.

5. Cell culture

Chinese hamster ovary (CHO-K1) cells (Russian Cell Culture Collection, Institute of Cytology, St. Petersburg, Russia) were maintained in Dulbecco's Modified Eagle Medium: Nutrient Mixture F12 (DMEM/F12; Gibco, Carlsbad, CA, USA) media supplemented with fetal bovine serum (FBS , 10 % v/v; Gibco, Carlsbad, CA, USA), L-glutamine (2 mM; Capricorn Scientific, Germany), and penicillin/streptomycin (100 U/mL;Invitrogen, Thermo Fisher Scientific, USA). Cultures were incubated at 37 °C in a humidified atmosphere with 5 % CO_2. The cells were seeded in tissue culture flasks 25 cm^2 (Orange scientific, Belgium) and sub-cultured using trypsin-EDTA (Thermo Fisher Scientific, USA) three times a week. For microscopy, the cells (1×10^5) were seeded on glass bottom dishes 35 mm (Ibidi, Gräfelfing, Germany) and incubated for 2 days until reaching a confluence of ca. 70–90 %. The aqueous **Pt1@PCL-*b*-PEG** micellar solutions (2 mg/mL) were filtered through 0.22 μm PTFE filters to prevent possible bacterial contamination. After removal of the growth media, the cells were treated with a new portion of DMEM/F12 containing the probe solution at a final concentration of polymer nanoparticles of 0.3 mg/mL. After incubation with the probe, confocal microscopy and PLIM measurements were performed at 37 °C in a humidified atmosphere containing 5 % CO_2. Hypoxia was induced by blowing the nitrogen-air mixture at preset volume ratio above the cell monolayer for ca. 30 min [S2,S3]. These conditions were maintained by using Stage Top Incubator Tokai HIT (Fujinomiya, Japan).

6. Cell viability (MTT) assay

The toxicity of the phosphorescent micelles, empty and loaded with **Pt1**, was evaluated using MTT assay [S4]. The CHO-K1 cells were plated in 96-well flat-bottom plates ("TPP Techno Plastic Products AG", Trasadingen, Switzerland) at a concentration of 10^4 cells per well. The cells were incubated for 24 hours at 37 °C, 5 % CO_2 in standard culture media (DMEM/F12; 10 % FBS; 100 U penicillin/streptomycin). The samples of luminescent probes in aqueous solution were added to the cells at concentrations 0.05–0.3 mg/mL. Six replications were performed for each concentration. After 24 hours of incubation with the samples, the medium in each well was replaced with 100 μL of fresh culture medium containing 0.25 mg/mL of MTT (3(4,5-dimethyl-2-thiasolyl)-2,5-diphenyl-2H-tetrasole bromide). After 2 hours of incubation, MTT media was replaced by 100 μL of DMSO (Helicon, Russia) to dissolve formazan crystals. After 15–20 minutes of incubation at 37 °C, the plates were shacked thoroughly using an orbit plate shaker and then the absorbance was read at 570 nm wavelength by using a SPECTROstar Nano microplate reader (BMG LABTECH, Ortenberg, Germany). Viability was calculated as the ratio of sample's optical density to the control one. The results are shown as mean ± standard deviation of not less than 6 repetitions.

Part 2. Supplementary Figures and Tables

Table S1. Characterization of **PCL-*b*-PEG** samples compositions by ^{1}H NMR and GPC.

Sample	[PEG]:[PCL] ^{a}	M_w, g/mol ^{b}	$Đ$ ^{b}
Sample #1	4.4:1.0	15000	1.33
Sample #2 (starting)	3.8:1.0	10800	1.41
Sample #2 ('purified') ^{c}	4.5:1.0	11500	1.31
Data by the Manufacturer	2.6:1.0 ^{d}	11800 ^{d}	1.18

^{a} Measured by ^{1}H NMR; ^{b} Measured by GPC; ^{c} The 'purified' Sample #2 was separated from precipitate three times by the protocol described in Materials and Methods (see Main text); ^{d} The expected value calculated from the data provided by the Manufacturer.

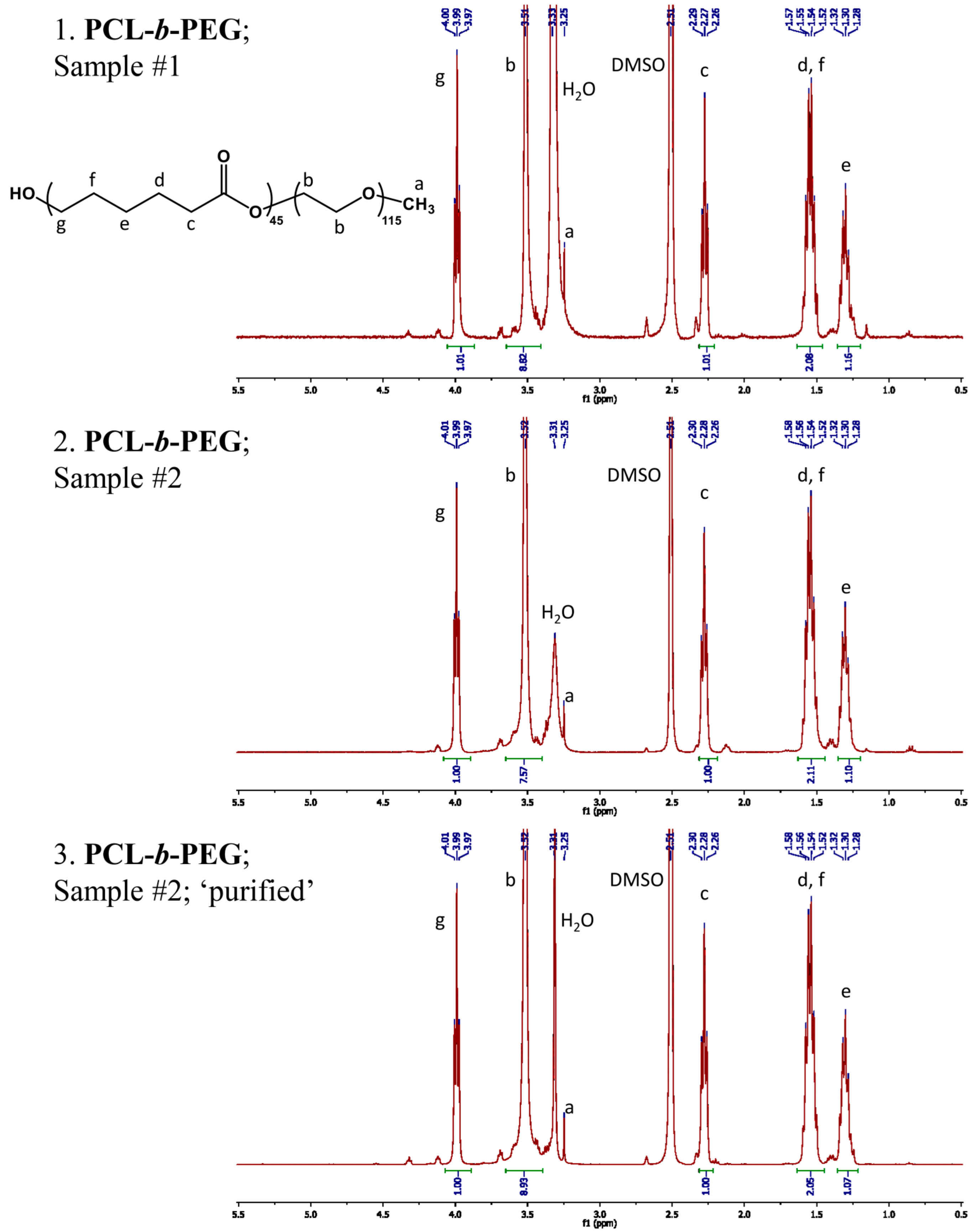

Figure S1. ^{1}H NMR spectra of **PCL-*b*-PEG** samples used in the study.

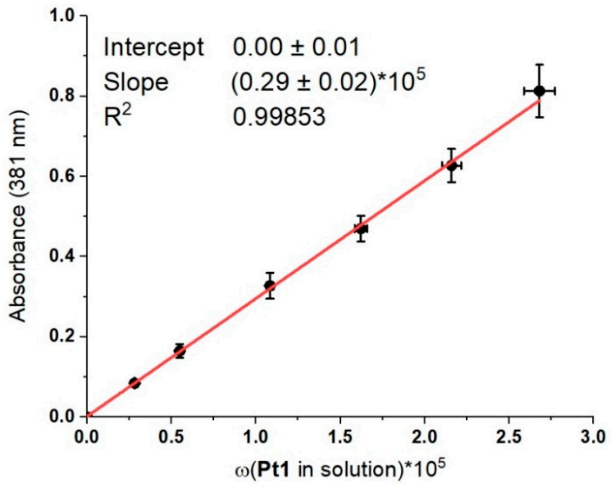

Figure S2. Calibration curve of **Pt1** absorption in DMF at 381 nm in the presence of 0.25 mg/mL of **PCL-*b*-PEG**.

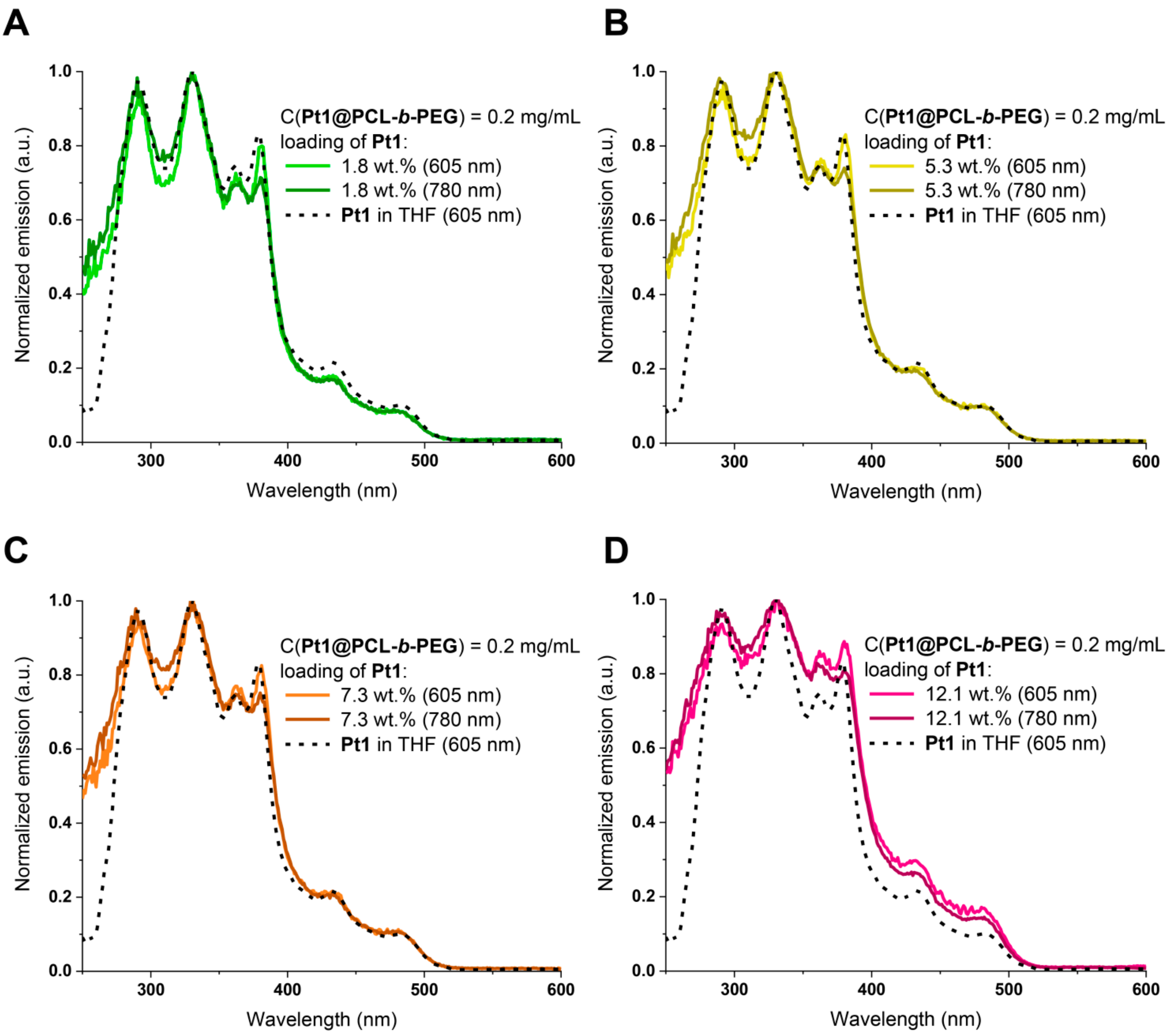

Figure S3. Normalized excitation spectra of **Pt1@PCL-*b*-PEG** loaded by 1.8 (A), 5.3 (B), and 7.3 (C), and 12.1 (D) wt.% of **Pt1** complex detected at 605 and 780 nm.

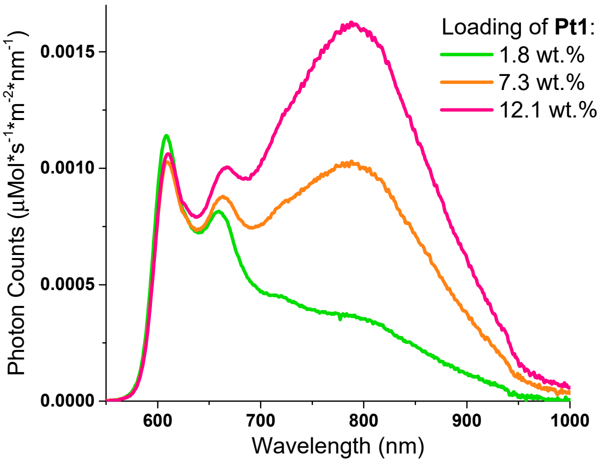

Figure S4. Non-normalized emission spectra of **Pt1@PCL-*b*-PEG** micellar dispersions in water. Excitation at 365 nm. C(**Pt1@PCL-*b*-PEG**) = 0.2 mg/mL.

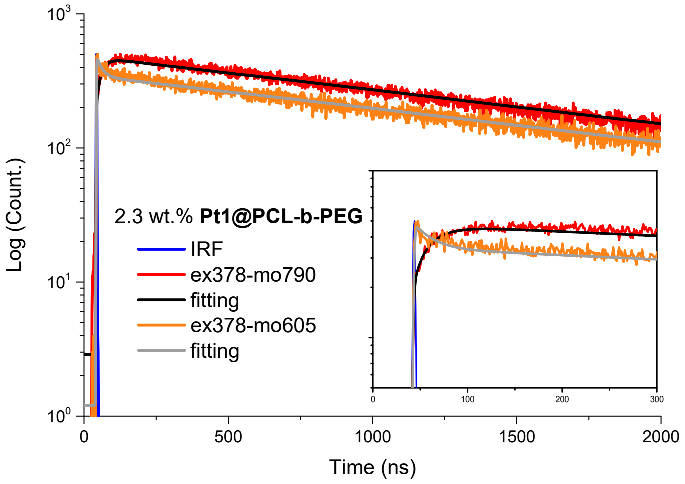

Figure S5. The emission rise and decay kinetics of **Pt1@PCL-*b*-PEG** (2.3 wt.% of **Pt1**) in aerated water dispersion at room temperature monitored at 605 nm and 790 nm. Black and gray curves represent the resulting fittings. Note that the fast decay of 19.5 ns monitored at 605 nm is identical with the rise component (19.5 ns) of the 790 nm, indicating a precursor-successor type kinetic relationship (see the main text for detail).

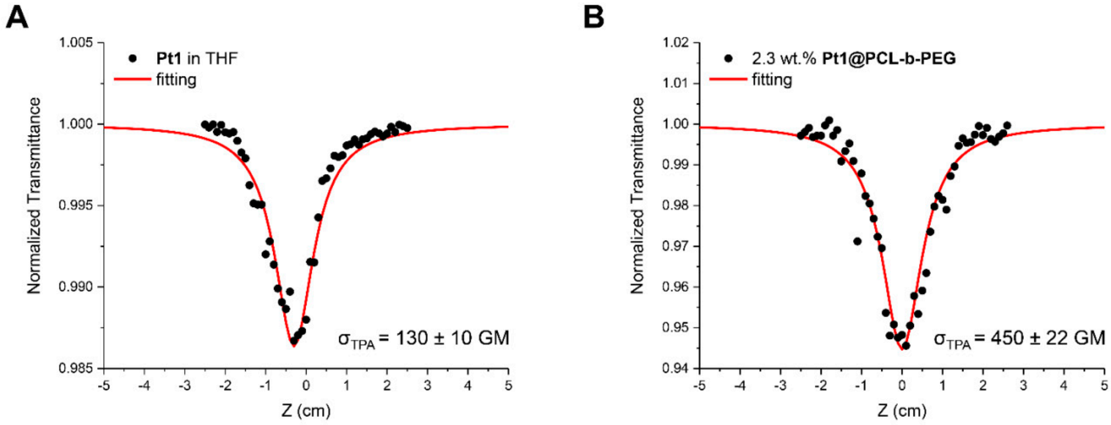

Figure S6. The Z-scan experimental data of **Pt1** in THF (A) and **Pt1@PCL-*b*-PEG** (2.3 wt.% of **Pt1**) in aerated water (B) at room temperature (circles). Solid lines are the result of a fit to the data points based on eqs (2)-(4).

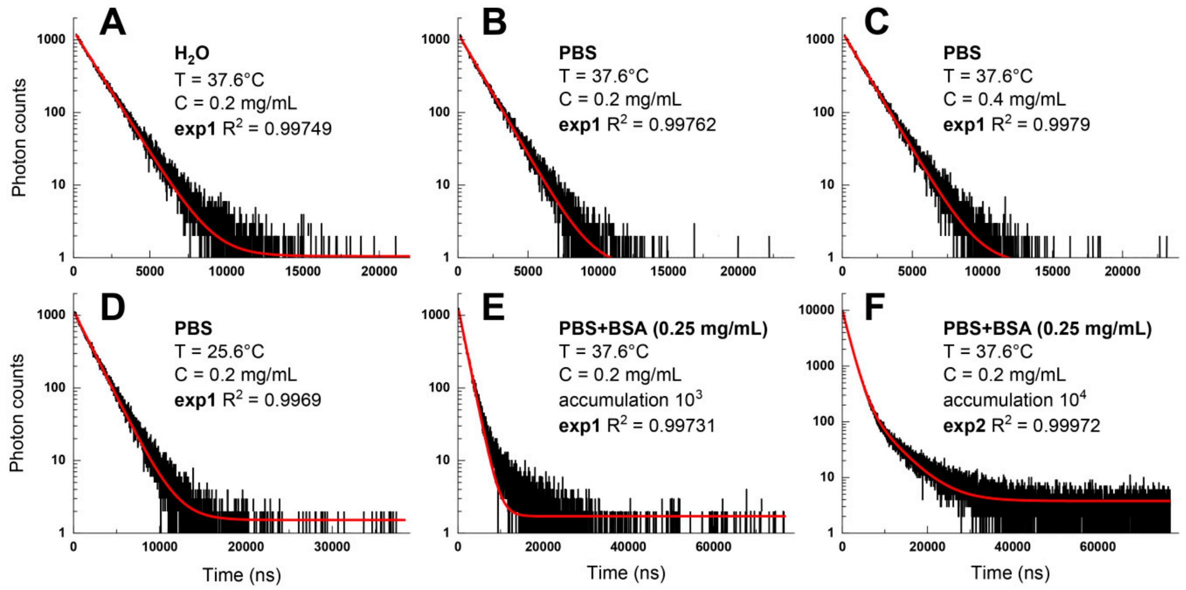

Figure S7. Luminescence decay curves of **Pt1@PCL-*b*-PEG** (2.3 wt.%) in aerated dispersions. A. water; 0.2 mg/mL; 37.6 °C. B. PBS; 0.2 mg/mL; 37.6 °C. C. PBS; 0.4 mg/mL; 37.6 °C. D. PBS; 0.2 mg/mL; 25.6 °C. E. BSA in PBS (0.25 mg/mL BSA); 0.2 mg/mL; 37.6 °C; counting to 10^3 photons; single exponential fit. F. BSA in PBS (0.25 mg/mL BSA); 0.2 mg/mL; 37.6 °C; counting to 10^4 photons; double exponential fit. Red curves represent the resulting mono- (A–E) or biexponential (F) fittings. Lifetimes calculated via the corresponding fittings are presented in Table 2 (main text; A–E except F). Detection wavelength: 605 nm.

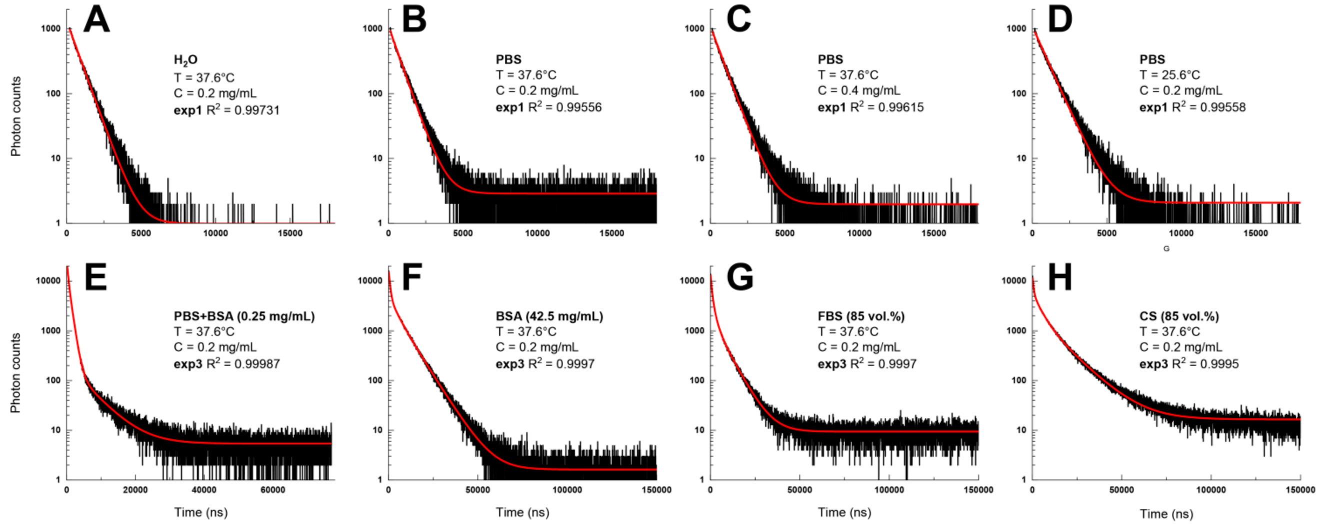

Figure S8. Luminescence decay curves of **Pt1@PCL-*b*-PEG** (12.1 wt.%) in aerated dispersions. A. water; 0.2 mg/mL; 37.6 °C. B. PBS; 0.2 mg/mL; 37.6 °C. C. PBS; 0.4 mg/mL; 37.6 °C. D. PBS; 0.2 mg/mL; 25.6 °C. E. BSA in PBS (0.25 mg/mL BSA); 0.2 mg/mL; 37.6 °C; F. BSA in PBS (42.5 mg/mL BSA); 0.2 mg/mL; 37.6 °C; G. Fetal Bovine Serum (85 vol. %); 0.2 mg/mL; 37.6 °C; H. Calf Serum (85 vol. %); 0.2 mg/mL; 37.6 °C. A–D: counting to 10^3 photons; single exponential fit; E-H: counting to 10^4 photons; triexponential fit. Red curves represent the resulting fittings. Lifetimes calculated via the corresponding fittings are presented in Table 1 (main text; A–D) or in Table S3 (F–H). Detection wavelength: 605 nm.

Table S2. Lifetimes of **Pt1@PCL-*b*-PEG** dispersion (12.1 wt.% of **Pt1**) in aerated water at 37.6 °C collected at different wavelengths.

Wavelength, nm	τ_{major}, µs [a]	Relative contribution of τ_{major}, %	τ_{minor}, µs [a]
605	0.69 ± 0.04	92	0.07 ± 0.004
665	0.73 ± 0.04	95	0.16 ± 0.08
725	0.71 ± 0.04	95	2.34 ± 0.12

[a] Decay curves were fitted by biexponential decay.

Table S3. Lifetimes of **Pt1@PCL-*b*-PEG** (12.1 wt.% of **Pt1**) in complex biological media.

Dispersion media	C, mg/mL	T, °C	pO_2, mmHg	τ, µs [a]
BSA, 42.5 mg/mL	0.2	37.6	154 ± 1	5.30 ± 0.05
			23.2 ± 0.6	8.97 ± 0.05
			10 ± 2	9.64 ± 0.06
Fetal Bovine Serum, 85 vol.%	0.2	37.6	146 ± 3	3.9 ± 0.3
			109.5 ± 0.7	4.70 ± 0.3
			18 ± 4	7.08 ± 0.4
Calf Serum, 85 vol.%	0.2	37.6	1.24 ± 0.06	8.13 ± 0.4

[a] Decay curves were fitted by triexponential decay with $\tau = (A_1\tau_1^2 + A_2\tau_2^2 + A_3\tau_3^2)/(A_1\tau_1 + A_2\tau_2 + A_3\tau_3)$.

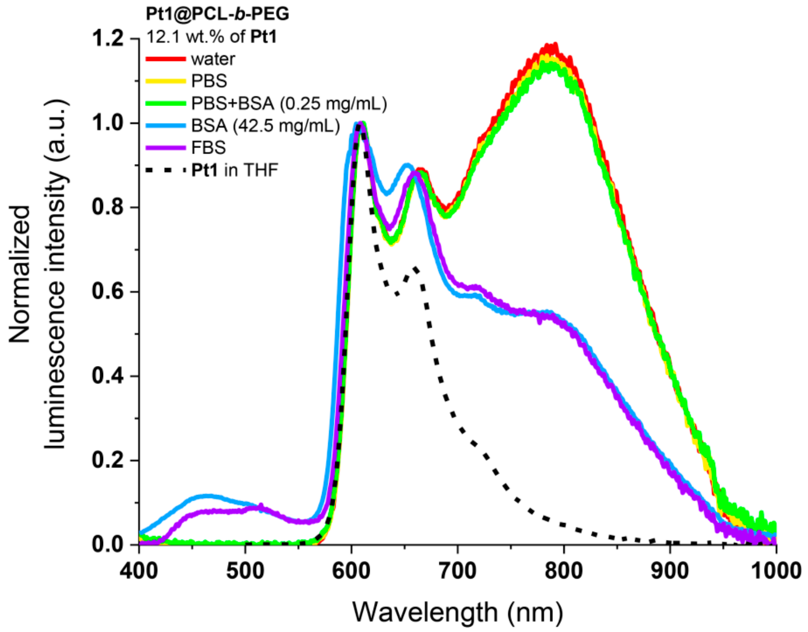

Figure S9. Normalized emission spectra of **Pt1@PCL-*b*-PEG** (12.1 wt.% of **Pt1**) micellar dispersions in water, PBS, in the presence of BSA (0.25 and 42.5 mg/mL) and Fetal Bovine Serum. Excitation at 365 nm. Dashed line represents emission spectrum of **Pt1** in THF.

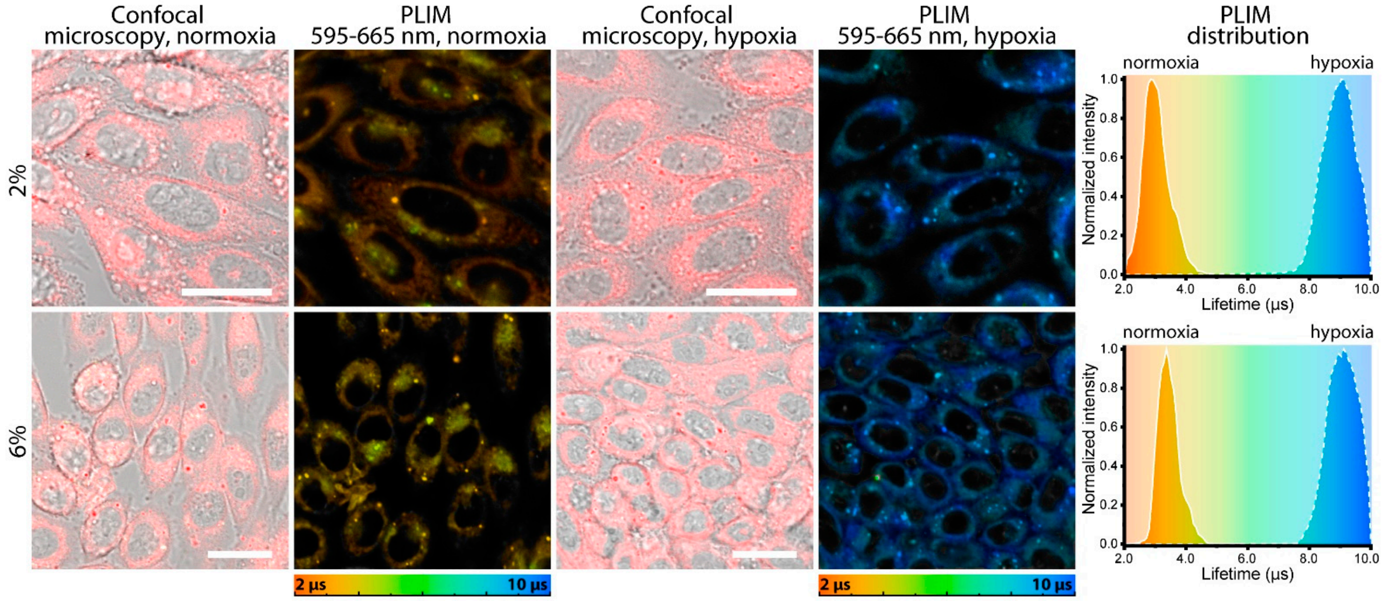

Figure S10. Confocal/DIC and PLIM microphotographs of CHO-K1 cells incubated with 0.3 mg/mL **Pt1@PCL-*b*-PEG** with 2 wt.% **Pt1** (top) and 6 wt.% **Pt1** (bottom) for 24 h under normoxia and hypoxic conditions. Lifetime distributions across the whole images for normoxia (solid line, 2–5 μs) and hypoxia (dashed line, 6–10 μs) are showen in the right panel. Excitation: 405 nm; Detection: 663–738 nm (confocal microscopy; left column); 595–665 nm (PLIM; middle column). Scale bar: 20 μm.

Supporting References

S1. Solomatina, A.I.; Galenko, E.E.; Kozina, D.O.; Kalinichev, A.A.; Baigildin, V.A.; Prudovskaya, N.A.; Shakirova, J.R.; Khlebnikov, A.F.; Porsev, V.V.; Evarestov, R.A.; Tunik, S.P. Non-Symmetric [Pt(C^N*N′^C′)] Complexes: Aggregation Induced Emission in Solid State and in Nanoparticles Tuned by Ligand Structure. *Chem. Eur. J.* **2022**, *28*, submitted.

S2. Conway, J.R.W.; Warren, S.C.; Herrmann, D.; Murphy, K.J.; Cazet, A.S.; Vennin, C.; Shearer, R.F.; Killen, M.J.; Magenau, A.; Mélénec, P.; et al. Intravital Imaging to Monitor Therapeutic Response in Moving Hypoxic Regions Resistant to PI3K Pathway Targeting in Pancreatic Cancer. *Cell Rep.* **2018**, *23*, 3312–3326, doi:10.1016/j.celrep.2018.05.038.

S3. Kritchenkov, I.S.; Elistratova, A.A.; Sokolov, V. V.; Chelushkin, P.S.; Shirmanova, M. V.; Lukina, M.M.; Dudenkova, V. V.; Shcheslavskiy, V.I.; Kalinina, S.; Reeß, K.; et al. A Biocompatible Phosphorescent Ir(III) Oxygen Sensor Functionalized with Oligo(Ethylene Glycol) Groups: Synthesis, Photophysics and Application in PLIM Experiments. *New J. Chem.* **2020**, *44*, 10459–10471, doi:10.1039/D0NJ01405B.

S4. Mosmann, T. Rapid Colorimetric Assay for Cellular Growth and Survival: Application to Proliferation and Cytotoxicity Assays. *J. Immunol. Methods* **1983**, *65*, 55–63, doi:10.1016/0022-1759(83)90303-4.
